# *Crotalaria verrucosa* Leaf Extract Mediated Synthesis of Zinc Oxide Nanoparticles: Assessment of Antimicrobial and Anticancer Activity

**DOI:** 10.3390/molecules25214896

**Published:** 2020-10-23

**Authors:** Siva Sankar Sana, Divya Vishambhar Kumbhakar, Akbar Pasha, Smita C. Pawar, Andrews Nirmala Grace, Raghvendra Pratap Singh, Van-Huy Nguyen, Quyet Van Le, Wanxi Peng

**Affiliations:** 1 Department of Materials Science & Nanotechnology, Yogi Vemana University, Kadapa 516005, India; sanasivasankar1@gmail.com; 2 Department of Genetics & Biotechnology, Osmania University, Hyderabad 500007, India; kumbhakardivya@gmail.com (D.V.K.); akbar.pasha786@gmail.com (A.P.); smita.prof@gmail.com (S.C.P.); 3 Centre for Nanotechnology Research, Vellore Institute of Technology, Vellore 632014, India; andrewsnirmalagrace@gmail.com; 4 Department of Research and Development, Biotechnology, Uttaranchal University, Dehradun 248007, India; singh.dr.raghvendra@outlook.com; 5 Department for Management of Science and Technology Development, Ton Duc Thang University, Ho Chi Minh City 700000, Vietnam; 6 Faculty of Applied Sciences, Ton Duc Thang University, Ho Chi Minh City 700000, Vietnam; 7 Institute of Research and Development, Duy Tan University, Da Nang 550000, Vietnam; 8 Henan Province Engineering Research Center for Biomass Value-added Products, School of Forestry, Henan Agricultural University, Zhengzhou 450002, China

**Keywords:** ZnO NPs, *C. verrucosa*, characterization, antimicrobial activity, anti-cancerous potentiality

## Abstract

In this work, we present an ecofriendly, non-hazardous, green synthesis of zinc oxide nanoparticles (ZnO NPs) by leaf extract of *Crotalaria verrucosa* (*C. verrucosa*). Total phenolic content, total flavonoid and total protein contents of *C. verrucosa* were determined. Further, synthesized ZnO NPs was characterized by UV–visible spectroscopy (UV-vis), X-ray diffractometer (XRD), Fourier transform infra-red (FTIR) Spectra, transmission electron microscope (TEM), and Dynamic light scattering (DLS) analysis. UV-vis shows peak at 375 nm which is unique to ZnO NPs. XRD analysis demonstrates the hexagonal phase structures of ZnO NPs. FTIR spectra demonstrates the molecules and bondings associated with the synthesized ZnO NPs and assures the role of phytochemical compounds of *C. verrucosa* in reduction and capping of ZnO NPs. TEM image exhibits that the prepared ZnO NPs is hexagonal shaped and in size ranged between 16 to 38 nm which is confirmed by DLS. Thermo-gravimetric analysis (TGA) was performed to determine the thermal stability of biosynthesized nanoparticles during calcination. The prepared ZnO NPs showed significant antibacterial potentiality against Gram-positive (S. aureus) and Gram-negative (*Proteus vulgaris*, *Klebsiella pneumoniae*, and *Escherichia coli*) pathogenic bacteria and SEM image shows the generalized mechanism of action in bacterial cell after NPs internalization. In addition, NPs are also found to be effective against the studied cancer cell lines for which cytotoxicity was assessed using MTT assay and results demonstrate highest growth of inhibition at the concentration of 100 µg/mL with IC_50_ value at 7.07 µg/mL for HeLa and 6.30 µg/mL for DU145 cell lines, in contrast to positive control (*C. verrucosa* leaf extract) with IC_50_ of 22.30 µg/mL on HeLa cells and 15.72 µg/mL on DU145 cells. Also, DAPI staining was performed in order to determine the effect on nuclear material due to ZnO NPs treatment in the studied cell lines taking leaf extract as positive control and untreated negative control for comparison. Cell migration assay was evaluated to determine the direct influence of NPs on metastasis that is potential suppression capacity of NPs to tumor cell migration. Outcome of the synthesized ZnO NPs using *C. verrucosa* shows antimicrobial activity against studied microbes, also cytotoxicity, apoptotic mediated DNA damage and antiproliferative potentiality in the studied carcinoma cells and hence, can be further used in biomedical, pharmaceutical and food processing industries as an effective antimicrobial and anti-cancerous agent.

## 1. Introduction

Nanoparticles (NPs) are the engineered assemblage of materials having nanoscale dimensions with a size distribution range of 1–100 nm diameters as defined by the National Nanotechnology Initiative (NNI), with unique physicochemical properties due to their nanosize, hydrophilic and hydrophobic nature, functional moieties on surface, high surface to volume ratio and aggregation properties [1]. Nanoparticles permit precision engineering to track and regulate their interaction with biological systems and hence nanotechnology integrated with biology is now very much involved in the creation of modern and more effective antimicrobial and anticancerous agents. Metal oxide nanoparticles provide promising and wide perspectives for biomedical fields through the production of nanomaterials, notably antimicrobial, anticancerous drugs, gene delivery, cell imaging, and biosensing [2]. Bacterial infections are considered to be a significant health issue on a global level. Mutations in bacterial genes and multiple drug resistance (MDR) have raised challenges to treat MDR microorganisms because resistance drastically restricts therapeutic options and hence there is a need for effective antibacterial agents without adverse effects on treated cells. ZnO NPs are efficient multifunctional inorganic metal oxide NPs that have a wide energy gap of 3.37 eV with short wavelength and large exciton binding energy of 60 meV, is considered to be moderately toxic but is extremely reactive and sensitive with extreme absorbent potentiality [3]. ZnO NPs have been given considerable attention by varied applications in terms of their antimicrobial properties thereby reducing the problem of multi-drug resistance. Zinc is considered to be a significant trace element in tissues of the body, such as brain, muscle, bone, and skin, maintaining many physiological functions and without which few enzymes namely carbonic anhydrase, carboxypeptidase, and alcohol dehydrogenase become quiescent [4]. Being a central component of different enzymes, zinc is involved in body metabolism and plays a vital role in hematopoiesis, synthesis of proteins and nucleic acid [5,6,7]. While, the eukaryotes are found resistant to lower concentrations of zinc oxide NPs [8]. Interestingly, nanosized ZnO is classified and recognized as safe (GRAS) by the US Food and Drug Administration (FDA) [9].

Nanocrystals of ZnO are simple to produce, eco-friendly, non-toxic, biosafe, and bio-compatible, which renders them for perfect biological application [10]. The effect of the prepared nanoparticle on functions of biological system depends on its concentration, particulate size, exposure duration, concentration, and pH. Some antibacterial activities are documented for ZnO NPs showing its potential antimicrobial activity against several pathogenic microbes such as *Staphylococcus aureus*, *Bacillus subtilis*, *Pseudomonas aeruginosa*, *Bacillus megaterium*, *Staphylococcus lutea*, *Escherichia coli*, *Klebsiella pneumonia*, *Proteus vulgaris*, *Candida albicans*, *Listeria monocytogenes,* and *Aspergilis niger* [11,12]; proving that smaller sized NPs have stronger inhibitions [13,14]. ZnO NPs are also reported as attractive candidate for biomedical applications in terms of their unique characteristics such as relative easiness of growth, the capability to modify their features, their intrinsic cytotoxicity in contrast to cancer cells essentially osteoblast cancer cells, human bronchial epithelial cells (BEAS-2B), human alveolar adenocarcinoma cells, human hepatocytes, embryonic kidney cells [15,16,17,18], and their ability to use as chemotherapeutic medicines in cancer targeting molecules [19,20]. Cell migration, invasion and adhesion are pivotal steps in the development of cancer; in contrast, approaches to overcome metastasis via treatments are relevant. Using phytomediated ZnO NPs, we aim to explain in vitro migratory behavior of HeLa and DU145 carcinoma cell.

Various methods were employed for synthesizing ZnO NPs. Among them, green synthesis method is a one-step synthesis of nanoparticles that requires relatively low energy to initiate the reaction. It provides the flexibility to produce nanoparticles of different sizes and is preferred over chemical and physical methods to overcome the high cost and harmful usage of chemicals circumventing its harsh reduction and stabilization condition [21]. Considering this, several metal oxide nanoparticles were synthesized successfully by biological processes [22,23]. Various plant parts including roots, leaves, seeds, stems, and fruits were involved in NPs synthesis because their rich extracts are documented to act as both reducing and stabilizing agents [24,25,26,27]. Plant-based polyphenol compound has potential bioactive properties, including high antioxidant activity, cytotoxic and antiproliferative activities [28,29,30].

**Figure 1:**
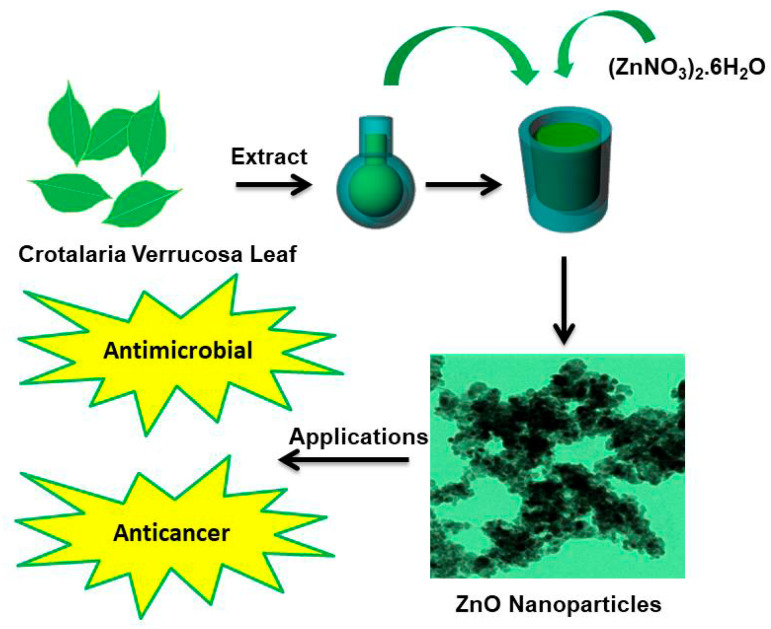
Schematic illustration of the biological synthesis of ZnO NPs using *Crotalaria verrucosa* leaf extract and its applications.

*Crotalaria verrucosa* L. plant belongs to family Papilionaceae, commonly known as blue rattle pod, Kilukiluppai, and Ghelegherinta, is a small shrub broadly distributed all over India, from the Himalayas to Ceylon [31]. This plant is generally cultivated as manure in improving soil quality for vegetation [32]. The leaf extract was used in traditional medicine against several diseases such as rheumatism, skin allergies, tetanus, salviations, biliousness, scabies, impetigo, dyspepsia, diarrhoea, jaundice, cardiac abnormalities, fever, dysentery, and leprosy [33,34,35,36] which is due to presence of bioactive compounds.. Previous literature stated that the leaf extract of *C. verrucosa* contains alkaloids, flavonoids, glycosides, tannins, steroids, polyphenolic compound, among others, and majorly alkaloids, flavonoids, tannins, and terpenoids reported to show significant cytotoxicity on cancer cell [37,38]. Ahmed et al. [39] manifested that leaf extract of *C. verrucosa* is effectively cytotoxic particularly against cervical HeLa cells due to (+)-Catechin hydrate and (−)-Epicatechin (tannins) compound which is responsible for its anticancer potentiality. Flavonoids due to presence of hydroxyl group act as antioxidants by preventing or neutralizing the free radical formation in the body [40,41]. Thus, investigation aimed to synthesize ZnO NPs using *C. verrucosa* to evaluate the antibacterial, cytotoxic and anticancerous potency. Therefore, the study has designed to synthesize and characterize ZnO NPs using *C. verrucosa* leaf extracts which would be eco-friendly, non-toxic and endowed with the antimicrobial activity against pathogens as well as anticancerous properties. The concept of our work is shown in Figure 1. The complex architecture of secondary plant metabolites are involved in nanoparticle synthesis and have shown to contribute in multi-target efficacy. Because of the complexities of cancer, a novel, multi-targeting bioactive compound is the need of the time. One potential cause may be the modulatory effect of the phytoconstituents on apoptosis which in comparison to normal cells is often blocked in cancer cells.

## 2. Results and Discussion

### 2.1. Phytochemicals of C. verrucosa

Ethanolic extract of *C. verrucosa* has been previously examined for its phytochemical tests and confirmed the presence of flavonoids and phytosterols in this plant. The aqueous leaf extract of *C. verrucosa* was subjected to the determination of phytochemicals such as polyphenols, flavonoids and proteins. The total phenolic content is estimated out to be 41 ± 1.1 GAE/gm and that of flavonoid content is found to be 8 ± 0.2 RE/gm. The total protein of the extract is estimated out to be 29 ± 0.9 mg/gm. The results are mean of three replicates. The hydroxyl groups of the secondary plant metabolite such as phenolic compounds and the flavonoids (such as flavonols, flavones, and condensed tannins) are reported to employ antioxidant activity both in vitro and in vivo, which help in promoting radical scavenging potential [42,43,44].The literature states that diverse group of plant extracts include secondary plant metabolites, phyto-proteins, flavonoids, polyphenolic compounds, alkaloids, polysaccharides, sugar compounds, aromatic amines, and amino acids have properties of both reducing (reduction of metal oxides to 0 valence metal NPs) and stabilizing agents [45,46,47]. *C. verrucosa* has previously reported for its several positive biological effects including antioxidative, anti-apoptotic, anti-aging, anticancer, and anti-inflammatory impacts, that is due to number of plant-based compounds such as polyphenolic and flavonoids [48]. Flavonoids are polyphenolic compounds possessing a structural backbone of diphenylpropane and have a strong capacity to function alike to antioxidants depending on their chemical structure; in addition, hydroxyl groups of flavonoids are essential for antioxidant and peroxy radical scavengers [41], thereby preventing lipid peroxidation [49]. Although phenolic compounds can serve as a free-radical eradicator and they actively participate in defense against oxidative stress. Phytoconstituents are traditionally used as medicine for which extraction and quantification of phytocompound is essential for the production of therapeutic drugs and is potential in providing direct treatment [50]. Hence, the characterized *C. verrucosa* leaf extract has represented their best candidature for NPs synthesis.

### 2.2. ZnO NPs Synthesis and its Characterization

Effective ZnO NPs is synthesized successfully using the leaf extract of *C. verrucosa* because of their exclusive phytochemicals and metabolite abundance. Presence of sunlight was also helpful for successful and effective green synthesis of ZnONPs. The phyto-molecules present in *C. verrucosa* extract are generally photosensitive flavoproteins and thus they enhance the reduction of metal ions for the effective synthesis and capping of NPs [51,52]. The color of the solution was turned from pale yellow to white, confirming ZnO NPs synthesis [46]. The synthesized ZnO NPs were reported to form white precipitate using plant extracts of different species like Rutagra veolens [53] and *Plectranthus amboinicus* [54], among others, which is in accordance with the results of our research. This visual color is the preliminary indication of NPs synthesis.

**Figure 2:**
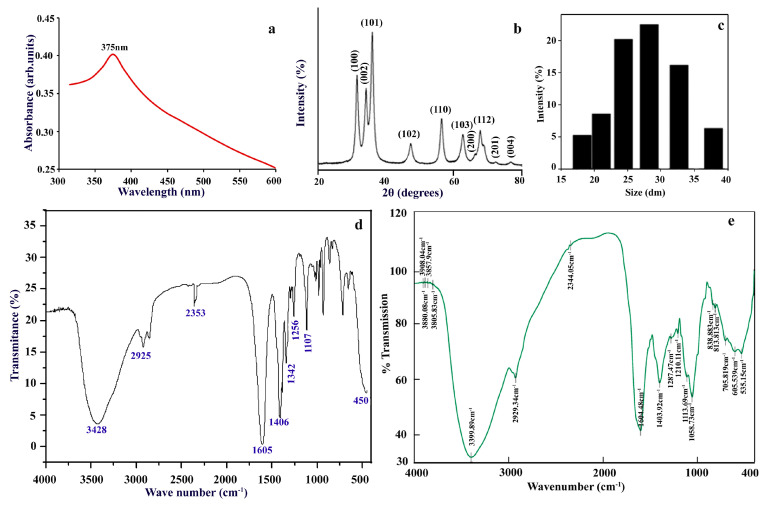
Characterization of green synthesized ZnO nanoparticles using *C. verrucosa* leaf. (**a**) UV-visible spectroscopy, (**b**) XRD, (**c**) Histogram showing DLS pattern of size distribution of ZnO-NPs, FTIR spectrum of (**d**) ZnO NPs and (**e**) leaf extract of *Crotalaria verrucosa.*

The UV visible absorption spectrum was performed to validate the biosynthesized NPs at the initial stage and evaluated in the range of 300–600 nm as shown in Figure 2a. The distinct peak of 375 nm is unique to ZnO nanoparticle formation due to their high excitation binding energy [55] confirming that leaf extract act as reducing agents during NPs formation. Bandgap increases with the decrease in particle size because of which blue shifting occurs in ZnO NPs compared to bulk ZnO [56].

The synthesized ZnO NPs have undergone X-ray diffraction to achieve crystallinity and to calculate average particle size. X-Ray Diffraction patterns of the biosynthesized ZnO NPs are presented in Figure 2b. XRD spectral analysis displays number of strong diffraction peaks corresponding to Bragg reflections with 2θ values of 31.94°, 34.28°, 36.20°, 47.49°, 56.54°, 62.86°, 66.43°, 67.89°, and 68.99°, that are indexed to (1 0 0), (0 0 2), (1 0 1), (1 0 2), (1 1 0), (1 0 3), (2 0 0), (1 1 2), and (2 0 1) planes respectively. This can be attributed to ZnO hexagonal wurtzite phase structures as per Joint Committee on Powder Diffraction Studies Standards (JCPDS file number: 36-1451) [57] and this confirms the crystalline structure of ZnO NPs. The crystallite size of ZnO NPs with the assistance of the highest peak by the Debye–Scherrer equation [58] obtained was calculated out to be 17.47 nm from a high-intensity peak. The broadening of a peak in a diffraction pattern suggests that the particles of the prepared NPs are in the nanometer range.

DLS is mainly used to measure the hydrodynamic diameter depending on particle Brownian movement of nanoparticle in suspension and the average hydrodynamic diameter measured by DLS histogram is 27 nm (Figure 2c) which varies from the size assessed from TEM and XRD. This difference in measured NP size with polydispersity index (pdi) value of 0.488 is attributed to the polydisperse nature of nanoparticles indicating non-uniform distribution of particles in nanosuspension and its occurrence in the form of aggregates which is evident from TEM images.

Substance-specific vibrations of the molecules contribute to the specific signals obtained by infrared spectroscopy. Spectroscopy of FTIR is a non-invasive and effective technique for investigating the role of functional atoms or biomolecules present in leaf extract which helps in the reduction and stabilization of NPs, also analyzes chemical bonds of NPs by its absorbance and transmittance value in the range of 400 to 4000 cm^−1^. FTIR spectrum of the biosynthesized ZnO NPs and aqueous leaf extract of *C. verrucosa* are illustrated in Figure 2d,e respectively, showing the broad peak at 3428 cm^−1^ for biogenic ZnO-NPs and 3399.89 cm^−1^ for leaf extract, which correspond to H bonded O–H stretching vibrations of polyphenols, alcohols [59,60]. FTIR spectral band exhibits weak absorption near 2925 and 2353 cm^−1^ (in case of ZnO NPs) and 2929.34 and 2344.05 cm^−1^ (for leaf extract), are attributed to C–H stretching vibration which depicts the presence of alkanes group. The peak at 1406 cm^−1^ (for ZnO NPs) and 1403.92 cm^−1^ (for leaf extract) refers to C-N vibration stretch in protein amide linkages [61,62]. The sharp absorption peak observed at 1603 cm^−1^ for ZnO and 1604.48 cm^−1^ for leaf extract are assigned to the N–H bending vibration of amine or amide groups [63]. The band observed at 1107 cm^−1^ (for ZnO NPs) and 1113.69 cm^−1^ (leaf extract) are assigned to C–O stretching alkoxy group or C-O-H in secondary and tertiary alcohols [64]. FTIR spectrum wavenumber of ZnO NPs (Figure 2d) confirms the presence of polyphenol, flavonoids, carbonyl, and aliphatic amine group of the plant extract are documented to act as both reducing and capping [65]. A sharp peak in the lower energy region was found at 450 cm^−1^ confirming Zn–O bond bending vibration as metal-oxygen are reported in the range of 400 to 600 nm [66]. Therefore, FTIR assessed for *C. verrucosa* mediated ZnO NPs (Figure 2d) corroborates with the result of FTIR spectrum of leaf extract, which shows that the organic compounds (phenols and flavonoid) of *C. verrucosa* still retain their original properties after calcination during ZnO synthesis and hence reduced the synthesized ZnO NPs.

**Figure 3:**
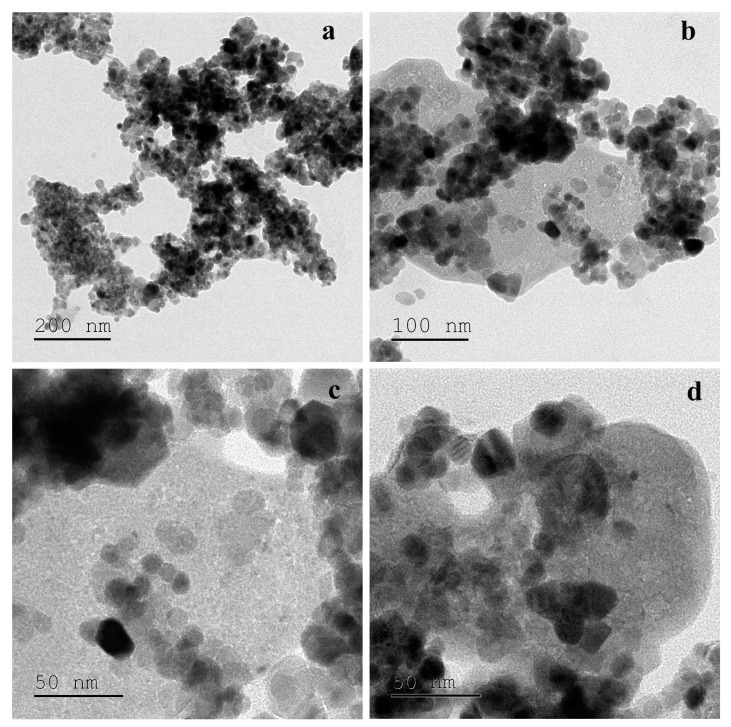
TEM image of nano-ZnO particles using *C. verrucosa* leaf extract at different scale: (**a**) 200 nm, (**b**) 100 nm, (**c**,**d**) 50 nm.

The TEM images of ZnO NPs at 200 nm (Figure 3a), 100 nm (Figure 3b) and 50 nm (Figure 3c,d) are shown. The TEM is a powerful tool for characterizing the ZnO NPs and was performed to understand the morphology and size of the NPs. TEM image of ZnO NPs shows a slight intense capping on the periphery of the nanoparticle surface due to biomolecules of aqueous leaf extracts showing that it acts as a reduction and capping agent. TEM confirms that NPs present in slightly aggregates form and show the size range of 16–38 nm particle sizes and the average particle size found out to be 27 nm and hexagonal shape (Figure 3).

**Figure 4:**
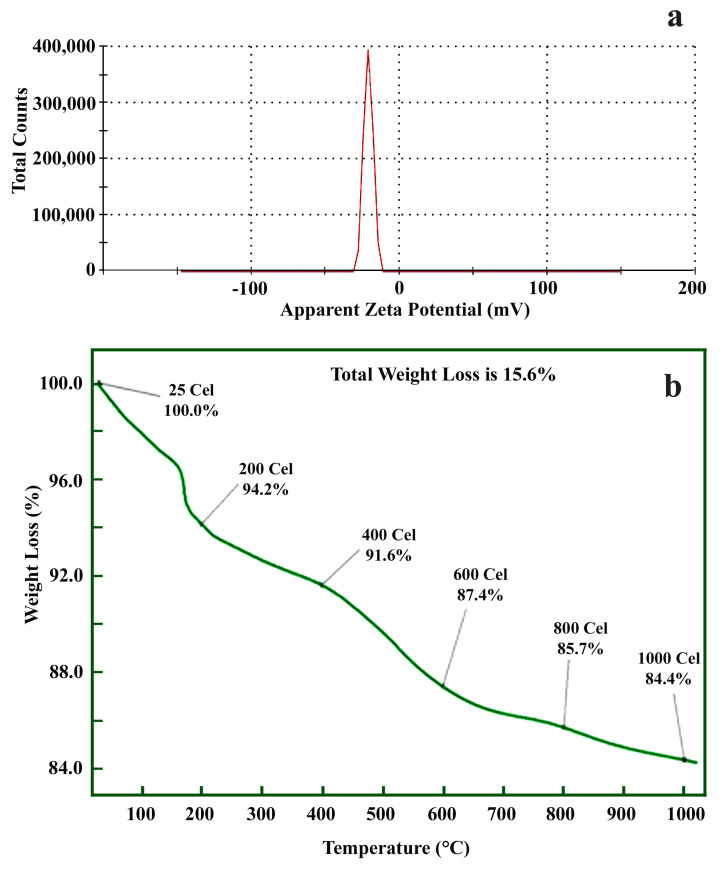
(**a**) Zeta potential analysis and (**b**) TGA analysis of green synthesized nano-ZnO particles using *C. verrucosa* leaf extract.

Zeta potential povides the zeta value which shows surface charge and stability of ZnO NPs (prepared using *C. verrucosa* leaf extract) was −21 mV (Figure 4a) demonstrating its stability. The hydroxyl group of *C. verrucosa* leaf extract is responsible for reduction of metal ion due to its strong binding affinity so that they can act as capping agent to avoid nanoparticle aggregation assuring its negative charge [38] and are in agreement with biosynthesized ZnO NPs from *S. album* leaf extract [67] and using *C. halicacabum* leaf extract [66]. TGA is used to assess the thermal stability of the synthesized NPs (20 mg) by subjecting the NPs to a temperature range of 0–1000 °C (Figure 4b) The reduction in weight measured at ~120 °C corresponds to the desertion of humidity. The actual reduction of weight was observed between ~340 and 550 °C, which corresponds to the elimination and decay of the very minute amount of organic compounds present in the sample during the green synthesis and decay of Zn and oxygen were measured at ~800 °C [68].

### 2.3. Antimicrobial Activity

**Figure 5:**
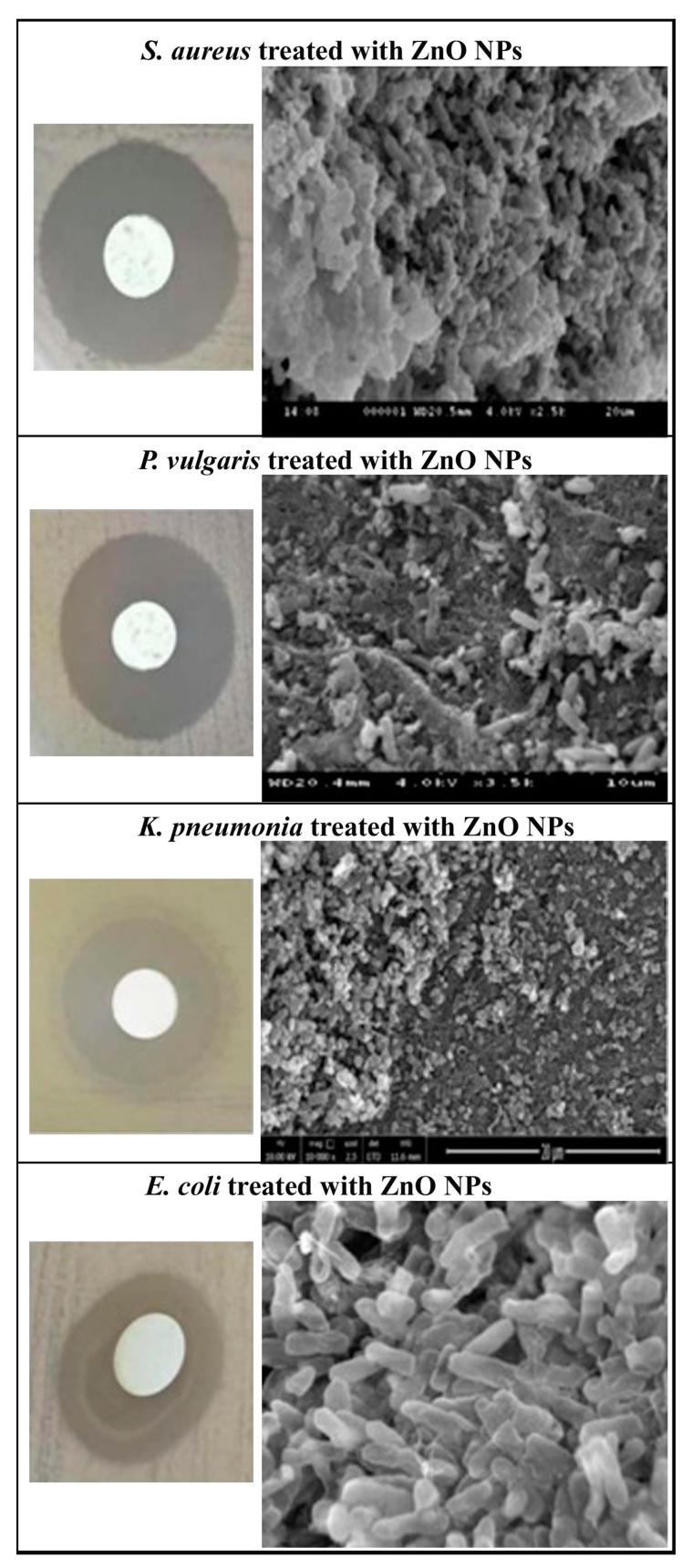
Zone of inhibition and SEM micrographs showing changes in morphology of *Staphylococcus aureus*, *Proteus vulgaris*, *Klebsiella pneumonia,* and *Escherichia coli* due to ZnO NPs treatments.

The zone of inhibition (in mm) showing the antibacterial effect of biosynthesized ZnO NPs against the tested bacterial strains and found that highest dose of ZnO NPs (100 µg/mL) shows effective antimicrobial activity in both gram positive and gram negative bacteria (Table 1 and Figure 5) alike to positive control, i.e., conventional antibiotic (20 µg/mL streptomycin) and proven that ZnO NPs can act as antibacterial agent as it is effective even at lower doses [69]. Due to the small size of NPs, it provides a large surface area which leads to more interaction between the NPs and the bacterial cells. The presence of the inhibition zone indicates the consequence and death of pathogens due to toxicity. ZnO NPs first interact with bacterial plasma membrane results subsequent entry of NPs into cytoplasm followed by the release of metal ions thereby disrupting membrane permeability eventually causes DNA damage leading to cell death [70,71]. Reporters suggested that Zn^2+^ ions are adhered to the biomolecules of the bacterial cell with electrostatic forces and are synchronized with lone pair of electron on nitrogen atom of protein and carbohydrate resulting in cessation of all important functions in bacteria [72]. SEM photomicrographs (as illustrated in Figure 5) clearly shows that the colony count of the studied bacteria were reduced in ZnO NPs treatments and distorted the shape and size of the cell wall. These results shows the leakage of the intracellular material irrespective of the thickness of bacterial cell wall and are in agreement with previous reports that zinc oxide nanoparticles can therefore be employed to prevent pathogenic microbial infections in the biological system [72]. Hydrogen peroxide generation is another reason for toxicity as it is capable of inducing oxidative stress such as singlet oxygen and hydroxyl radicals which in turn results in cell death [73,74].

### 2.4. Cell Viability Assay

**Figure 6:**
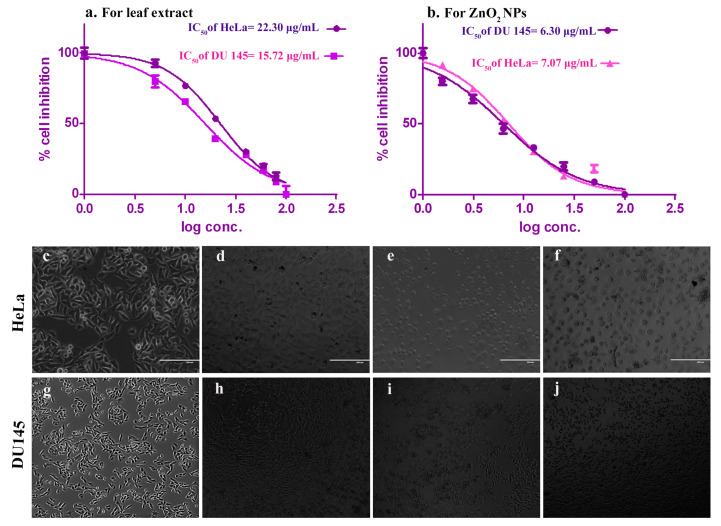
Cytotoxicity measured by MTT assay on HeLa and DU145 cell lines. (**a**) IC_50_ value of leaf extract treated cell lines, (**b**) IC_50_ value of ZnO NPs treated cell lines. Normal untreated negative control: (**c**) HeLa cell lines and (**g**) DU145 cell lines. Morphological changes due to lower dose of ZnO NPs on: (**d**) HeLa cell lines and (**h**) DU 145 cell lines. Morphological changes due to moderate dose of ZnO NPs on: (**e**) HeLa and (**i**) DU145 cell lines. Morphological changes due to higher dose of ZnO NPs on: (**f**) HeLa and (**j**) DU145 cell lines.

The cytotoxicity of the synthesized ZnO NPs against both the cell lines was screened by MTT assay at different concentrations upto 100 µg/mL for 24 h. Results exhibit dose-dependent inhibition curve at 50% with the inference of IC_50_ value of 7.07 µg/mL in HeLa cells and 6.30 µg/mL in DU145 (Figure 6b) compared to positive control (*C. verrucosa* leaf extract) with IC_50_ value of 22.30 µg/mL and 15.72 µg/mL for HeLa and DU145 cell lines (Figure 6a) respectively considering untreated negative controls (HeLa-Figure 6c, DU145-Figure 6g). It is clear that *C. verrucosa* leaf extract alone shows lesser cytotoxicity as inhibitory concentration value is more but when it was involved in ZnO NPs synthesis, the prepared NPs was found to show less inhibitory concentration in both the studied cell lines. ZnO NPs is reported to show selective destruction of carcinoma cell lines due to EPR effect [9]. Morphological changes due to ZnO NPs treated studied cell lines (HeLa and DU145) were observed under an inverted microscope (EVOS™ M5000, Thermo Fisher Scientific, Carlsbad, CA, USA) and photomicrographs validated the cytotoxicity in both carcinoma cells at higher (Figure 6f,j) and moderate doses of synthesized ZnO NPs (Figure 6e,i). Less morphological changes are observed in lower doses (Figure 6d,h) compared to that of control (Figure 6c,g). The biosynthesized ZnO NPs due to its semiconducting nature are reported to induce cytotoxicity in cancer cells by the generation of reactive oxygen species on the surface of the particle, the released Zn^2+^ ions are dissolved in culture media indicating direct interaction of NPs with a membrane of cancer cell resulting in oxidative stress thereby leading to the ultimate death of cancer cells [9]. Hanley et al. [75] recorded that ZnO NPs demonstrate 28–35 fold preferential toxicity in cancer cells relative to normal cells due to enhanced permeability and retention (EPR) and electrostatic interaction property of ZnO NPs.

### 2.5. DAPI Staining

**Figure 7:**
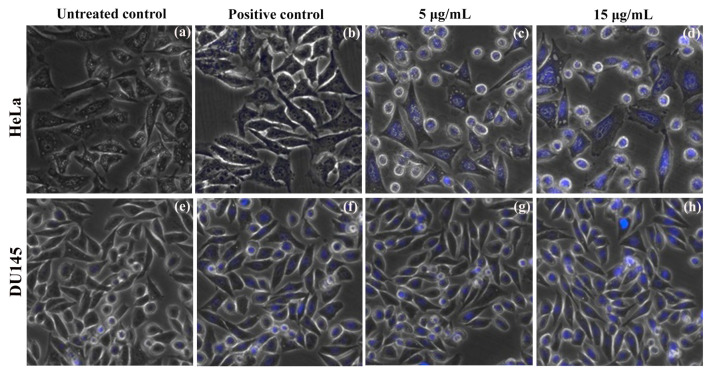
Nuclear staining using DAPI on HeLa and DU145 carcinoma cells observed at 20× magnification under fluorescent inverted microcope. Untreated negative controls: (**a**) HeLa, (**e**) DU145. 20 µg/mL leaf extract taken as positive controls for (**b**) HeLa and 15 µg/mL for (**f**) DU145. ZnO NPs treatment 5 µg/mL: (**c**) HeLa, (**g**) DU145; and 15 µg/mL ZnO NPs treatment: (**d**) HeLa, (**h**) DU145 cell lines.

DAPI (4′,6-diamidino-2-phenylindole) is a DNA binding fluorescent dye that strongly binds to AT-rich regions which gives blue fluorescence under fluorescence microscope. DAPI staining clearly depicts the morphological characteristics of nuclear damage linked with apoptosis in both HeLa and DU145 carcinoma cells, were investigated when treated with biosynthesized ZnO nanosuspension. The doses of ZnO NPs treated cells at 0 h and 24 h showed increased intensity of blue color fluorescence in a dose dependent manner in HeLa (Figure 7c,d) and DU145 (Figure 7g,h) compared with positive controls (Figure 7b for HeLa and Figure 7f for DU145) and untreated negative controls at 0 h and 24 h (Figure 7a for HeLa, Figure 7e for DU145). It is evident that the number of apoptotic cells increased considerably with the increase in ZnO NPs concentrations. There is a visible increase in blue fluorescence in treated cells in comparison to untreated and positive control cells indicating increase in intracellular zinc ion levels. Moreover, 15 μg/mL ZnO NPs treated HeLa (Figure 7d) and DU145 cells (Figure 7h) demonstrated higher fluorescence intensity than 5 μg/mL treatments (Figure 7c,g). Leaf extract (20 µg/mL, negative control) shows lesser nuclear damage in both HeLa (Figure 7b) and DU145 (Figure 7f) cells compared to treatments and more when compared with negative controls (Figure 7a,e). DAPI provides the study of nuclear morphology which is important for the evaluation of apoptosis, including the nucleus with condensed chromatin and chromatin fragmentation in early apoptosis. An increase in NPs concentration in cells contributes its attachment on cell surface followed by its uptake in cytoplasm resulting in cell death. ROS are produced in the cells in response to ZnO NPs exposure leading to free radical formation which reacts with the cell organelles to cause enzymatic changes, facilitates cellular content disorganization ultimately resulting in apoptosis [76,77].

### 2.6. In Vitro Scratch Wound Healing Assay

**Figure 8:**
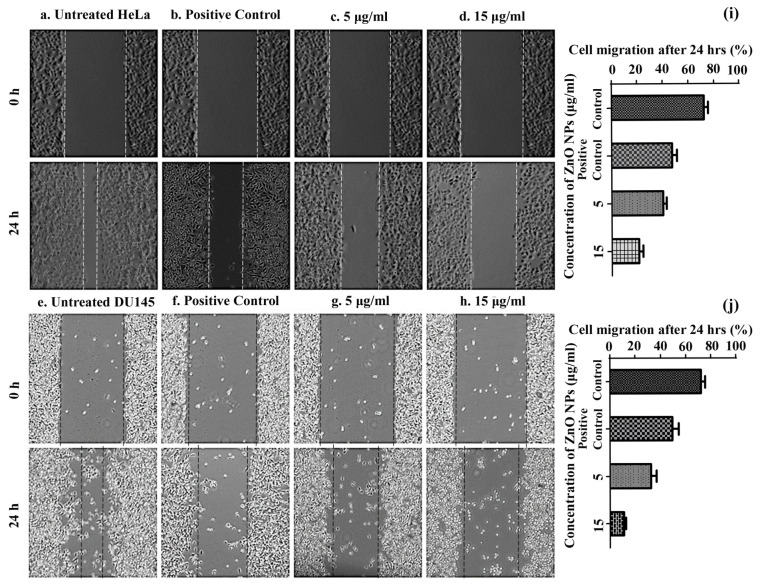
Analysis of HeLa and DU145 cell migration by in vitro scratch assay observed at 4× magnification under fluorescent inverted microscope and quantified using ImageJ software. (**a**) Untreated HeLa control at 0 and 24 h, (**e**) Untreated DU145 control at 0 and 24 h, aqeous leaf extract treated negative control at 0 and 24 h in (**b**) HeLa (**f**) DU145 cell lines. Low concentration of ZnO NPs treatment (5 µg/mL) at 0 and 24 h in (**c**) HeLa and (**g**) DU145 cell lines. High concentration of ZnO NPs treatment (15 µg/mL) at 0 and 24 h in (**d**) HeLa and (**h**) DU145 cell lines. (**i**) Relative inhibition of cell migration due to all treatments and controls in both cell lines after 24 h.

Results obtained from the assay clearly suggest the antiproliferative effect of the cell lines exerted by the prepared ZnO nanosuspension. Both the cells treated with concentration of 5 μg/mL (Figure 8c,g) and 15 μg/mL (Figure 8d,h) ZnO NPs and showed substantial migration of 40.45% (in 5 μg/mL) and 21.35% (in 15 μg/mL) for HeLa and 32.66% (in 5 μg/mL) and 11.17% (in 15 μg/mL) for DU145 cell lines after 24 h. Results shows lesser cell proliferation in treatments with respect to the positive controls (47.87% for HeLa-Figure 8b, 49.49% for DU145-Figure 8f) and untreated HeLa (Figure 8a) and DU145 (Figure 8e) cells with a migration rate of about 72.77% and 72.51% respectively. The relative rate of migration assessed by ImageJ software for both treatments and controls in both cell lines was presented in Figure 8i,j. These findings demonstrates that nanosized ZnO inhibits cell invasion and migration in proliferating studied cancer cells and are in accordance with biosynthesized ZnO NPs reports in other cell lines [9,78].

The reported in vitro studies have established that ZnO NPs selectively target and kills tumor cells due to its EPR effect [79,80,81]. Another feature of ZnO NPs is its electrostatic characteristic that aids in selective targeting purpose as the surface of ZnO NPs has a neutral hydroxyl group which plays role in surface charge behaviour. Since isoelectric pH of ZnO NPs is around 9–10 therefore, ZnO NPs exhibit positive charge in tissue fluid of cancer cells under physiological conditions [9,81]. Cancer cells have a high negatively charged anionic phospholipids on their outer membrane [82] that leads to an electrostatic connection between ZnO NPs and cancer cells facilitating selective targeting, uptake and eventually causing cytotoxicity.

### 2.7. Statistical Analysis

Experiments were performed in triplicates and the statistical analysis was carried out using SPSS 17.0 software. The difference of experimental data was analyzed by one-way ANOVA TEST. Data were shown as mean ± standard error. Statistical significance was considered as *p* < 0.05.

## 3. Materials and Methods

### 3.1. Chemicals and Reagents

Zinc nitrate [Zn(NO_3_)_2_·6H_2_O] was purchased from Merck, Mumbai, India. Meuller–Hinton agar (MHA), 3-[4,5-96dimethylthiazole-2-yl]-2,5-diphenyltetrazolium bromide (MTT), DAPI (4′,6-diamidino-2-phenylindole) and Dulbecco’s modified Eagle medium (DMEM) were obtained from Sigma-Aldrich and Himedia Laboratories. All the chemicals and reagents used in this study were graded with analytical reagents (AR) and the experiments involved in this study were done using milli-Q and sterile distilled water.

### 3.2. Preparation of Leaf Extract and Phytochemical Analysis

Leaves of *C. verrucosa* was collected from Sri Venkateswara University, Tirupathi, India and the plant species was identified and authenticated by an expert taxonomist Dr. Madhava Chetty, Assistant Professor at Department of Botany, Sri Venkateswara University, Tirupati. The collected fresh leaves of *C. verrucosa* were cleaned considerably with cool running tap water followed by milli-Q water. The washed leaves were then dried in the shade for 12–14 days until all the humidity had been lost, then grounded to powder in clean grinder. Ten grams of powdered leaves was weighed and boiled in Erlenmeyer flask containing 100 mL milli-Q for 10 min at 70 °C and allowed to cool at room temperature. The aqueous portion was then filtered via Whatman No. 1 filter paper and stored it at 4 °C in storage vials for further experimental usage. The bioactive compounds in the leaf extract were analyzed because these phytochemicals can act as stabilizing and reducing agents in the green synthesis of metal oxides. Remaining dried leaves were placed in an airtight vessel at normal temperature for consequent extract mediated NPs preparation.

#### 3.2.1. Total Phenolics

The total phenolic content in *C. verrucosa* leaf extract was estimated with the Folin–Ciocalteu method [83]. The extract of 140 µL (1 mg/mL) was mixed to react with 600 µL of Folin–Ciocalteu reagent (0.2 M) for 5min followed by the addition of 460 µL sodium carbonate (7.5%). The integral setup was incubated at 45 °C for 30 min followed by 1 h at room temperature in dark. The absorbance was measured at 650 nm (considering gallic acid as standard), calculated from calibration curve and outcome were presented as mg of gallic acid equivalents per gm (GAE/gm) of extract.

#### 3.2.2. Total Flavonoid Content

A colorimetric aluminium chloride (AlCl_3_) approach is used to estimate the total flavonoid contents of crude extract following Chang et al. [84]. Briefly, 25 µL (1 mg/mL) of the extract was added to 75 mL of methanol and the final volume was made upto 2 mL with distilled water. To this, 300 µL of 5% sodium nitrate and 300 µL of 10% AlCl_3_ were added followed by incubation of 10 min. Then 2 mL of 1 mol/L NaOH was added to the solution and final volume was adjusted upto 5 mL by using distilled water and allowed the solution to incubate for 40 min at room temperature and the OD was recorded at 510 nm. Results calculated from a calibration curve taking rutin as standard, and values were expressed as mg of Rutin equivalents per gram (mg RE/gm) of extract.

#### 3.2.3. Determination of Proteins

Total proteins in leaf extract were measured using the Biuret test by treating the extract with 1 mL (10%) of sodium hydroxide solution followed by heating. A drop of copper sulfate solution (0.7%) was mixed with the solution and incubated at room temperature for 30 min leading to the formation of purplish violet color and the OD was measured at 540 nm. Bovine serum albumin (BSA) was kept as standard and results are given as mg of BSA per gm of the sample.

#### 3.2.4. FTIR Spectrum Analysis of *C. verrucosa* Leaf Extract

Leaves of *C. verrucosa* were weighed and mixed with KBr salt forming a compressed pellet and was examined in FTIR spectroscope. Results were recorded using Perkin Elmer FTIR Frontier Spectrophotometer in the range of 4000–400 cm^−1^.

### 3.3. Green Synthesis of ZnO NPs

About 20 mL of *C. verrucosa* leaf extract (1 mg/mL) was taken in 250 mL Erlenmeyer flask and 2 gm of Zn(NO_3_)_2_·6H_2_O was added. For the purpose, 20 mL of plant extract was heated at 60 °C under continuous stirring in the presence of sunlight, followed by the addition of 2 gm of zinc nitrate. The mixture of the solution was continuously stirred until yellow color paste was obtained. The precipitate was calcinated at 400 °C for 3 h. Finally, white powder was obtained and used it for further analysis [85].

### 3.4. Characterization of Biosynthesized ZnO NPs

The biosynthesized ZnO NPs was monitored and characterized using UV–visible Spectrophotometer (Model 3092, Lab India, Mumbai, India.) The resulting nano-powder was resuspended in e sterile de-ionized water for UV–Vis spectral analysis in the wavelength range of 200–600 nm. Fourier transform infra red (FTIR) spectroscopy of ZnO NPs synthesized using *C. verrucosa* leaf extract was done to classify the different phytochemical components or the functional groups that are involved in the reduction and stabilization of nanoparticles. Briefly, the ZnO nanopowder (2 mg) has been combined with 200 mg of FTIR graded Potassium bromide and packed into a pellet for characterization. The sample pellets were put in the sample holder and the spectrum of FTIR was recorded using Perkin Elmer FTIR Frontier Spectrophotometer with the Attenuated Total Reflectance technology with a resolution of 4 cm^−1^ in 400 to 4000 cm^−1^ range. X-ray diffraction spectroscopic (XRD) analysis of dried ZnO NPs was performed using RIGAKU smart lab X-ray diffractometer to determine purity and quantitative analyses of various forms including the crystalline size of the synthesized nanopowder. Morphology, size and crystalline characteristics of NPs were obtained using a transmission electron microscopy (TEM). ZnO nanopowder was suspended and diluted by sterile deionized water to obtain turbid nanosuspension and was sonicated for 15 min. The nanosuspension was coated onto a copper grid followed by drying, was further analyzed by TEM JEOL 3010 at an acceleration voltage of 200 kV. Dynamic light scattering (DLS) determines the particle size and size distribution of the synthesized NPs using Malvern Zetasizer Nano ZS, Malvern, UK. The average particle dimensions of nanoparticle were analyzed using dynamic light scattering (DLS), which is based on a laser diffraction process with several scattering techniques. The prepared NPs are scattered and dispersed in deionized water followed by ultra-sonication. Zeta potential analyzer (Malvern Zetasizer Nano ZS, Malvern, UK) was used to determine the surface charges of the synthesized nanosized ZnO based on direction and velocity of particles in electric field, which provides insights into the stability ZnO NPs. Thermo-gravimetric analysis (TGA) has been conducted to determine thermal stability of biosynthesized NPs. The samples have been analyzed under nitrogen atmosphere at temperatures of 0 to 1000 °C/min with a rise of 10 °C/min using TGA (Mettler, Toledo, India).

### 3.5. Antimicrobial Activity

Human pathogenic bacteria *Escherichia coli* (MTCC 443), *Staphylococcus aureus* (MTCC 96), *Proteus vulgaris* (MTCC 426), and *Klebsiella pneumonia* (MTCC 432) were selected for the study and were collected from CSIR—Institute of Microbial Technology, Chandigarh, India. Pure cultures were subcultured and grown in Muller–Hinton (MH) broth at 37 °C for 24 h for further experiment. Each strain from the subcultured broth was swabbed uniformly using sterile cotton swabs onto the individual MHA Petri dishes. Three dilutions (25, 50, and 100 μg/mL) of ZnO NPs colloidal solution were tested against all the studied bacterial strains by using a disc diffusion method to evaluate the antimicrobial activity. The nanosuspension of all tested doses were added on sterile filter paper discs (6 mm in diameter—Himedia Lab) for antimicrobial susceptibility and left it to dry. Once the discs are dried, the individual discs of different concentrations were placed on top of the agar containing isolates and press it gently with a tweezer. Streptomycin (20 µg/mL) was kept as standard positive control and water treatment as negative control were taken for comparison against all the tested strains. The plates were incubated for 24 h under aerobic conditions at 37 °C and were examined after incubation. This experiment was conducted in triplicates to attain consistent outcomes.

#### Scanning Electron Microscope (SEM) Analysis

SEM studies were performed to confirm the morphological changes observed due to biosynthesized ZnO NPs followed by Gao et al. [86]. The microbial suspensions were incubated and grown in normal broth at 37 °C for 8 h and treated with 100 µg/mL ZnO NPs followed by incubation at 37 °C for 12 h. The microbes were pelleted by centrifugation (5,000 rpm for 5 min at 4 °C) and were washed with PBS (0.1 M, pH 7.4) three times for 10 min each, later fixed in 2.5% (*v*/*v*) glutaraldehyde in PBS (0.1 M, pH 7.4) for 2 h (4 °C). After washing and fixing, the microbial specimen were dehydrated using 50, 70, 80, 90, and 100% ethanol for 10 min each, then treated with tertiary butyl alcohol. The microbial specimens were ultimately dried using CO_2_ and coating was done with gold in an ion coater (2 min). The morphological change in the studied bacterial cells due to biogenic nanoparticle treatment was monitored with a SEM (S-3400 N, Hitachi, Ltd., Tokyo, Japan).

### 3.6. Cytotoxicity Assessment

#### 3.6.1. Cell Culture

Both HeLa and DU145 cell lines were procured from National Centre for Cell science (NCCS) Pune. The cells were inoculated and maintained in Dulbecco Modified Eagle’s Medium (DMEM) supplemented with 10% Fetal Bovine Serum (FBS) and 1% antibiotics (penicillin and streptomycin) followed by incubation in a humidified condition of 5% CO_2_ incubator (Thermo Scientific) at 37 °C for 24 h to allow the cells to grow. The cells were observed under an inverted microscope (EVOS™ M5000, Thermo Fisher Scientific, Carlsbad, CA, USA) to monitor the confluency and check the presence of contamination before initiating experiments. Both the incubated cells were passaged separately using trypsin–EDTA (0.25%) then centrifuged at 2000 rpm for 3 min and resuspended in DMEM media (1 mL) for further analysis.

#### 3.6.2. Cytotoxicity Assay

The MTT (3-(4,5-dimethyl thiazol-2yl)-2,5-diphenyl tetrazolium bromide) reduction assay was performed on cell lines to detemine the cytotoxicity due to prepared ZnO NPs. For analyzing the cell viability of both the studied carcinoma cells in vitro, 100 µL of respective cells (approximately 7500 cells/well) were seeded in each well of 96 well plates separately and incubated for 24 h under 5% CO_2_ at 37 °C so that the cells attach to the bottom of the each well. Stock solution (2 mg/mL) of the synthesized ZnO NPs was prepared and diluted to a range of 1:1 to 1:64 with culture media. Both the cells were treated separately with ZnO NPs in a dose dependent manner with highest concentration of 100 µg/mL and lower dose of 1.5625 µg/mL (1:1 to 1:64 dilutions) in the respective wells followed by incubation of the cells (37 °C, 5% CO_2_ incubator for overnight). *C. verrucosa* leaves extract (working solution of 1mg/mL was prepared from 40 mg/mL stock solution of leaf extract and diluted to the range of minimum dose of 5 µg/mL and maximum dose of 100 µg/mL) treated cells were taken as positive controls and untreated cells containing only DMEM medium were taken as negative control for both respective cells. MTT (20 µL/well of 5 mg/mL in PBS) was added to each well of overnight incubated cells (both treatments and controls) and again incubated at 37 °C for 4 h. The mitochondrial dehydrogenase reduce the yellowish water-soluble MTT to water-insoluble purple formazan crystals which was further solubilized using DMSO (100 µL) and kept in a shaker (30 min). The quantity of formazan crystal is directly proportional to the count of viable cells and absorbance was measured at 590 nm using a microplate reading spectrophotometer (Bio-Rad). Experiments were conducted in triplicates for the studied cell lines and growth inhibition was evaluated. Relative cell viability of NPs treated cells were calculated as: Mean OD test/control OD × 100 and their IC_50_ were calculated via Graph-pad Prism software.

#### 3.6.3. Morphological Assessment of Apoptotic Cells by DAPI Staining

Both the cultured HeLa and DU145 cells were treated with different doses of green synthesized ZnO NPs and leaf extract (positive control) to monitor the induced effect on cell nucleus using DAPI stain which allows the visualization of the condensed chromatin of the apoptotic cells. The cells were seeded in a 6 well plate (3 × 10^4^ cells/well) and incubated in CO_2_ incubator for 24 h at 37 °C. These overnight incubated cells were then treated with different concentration of ZnO NPs considering IC_50_ value (5 and 15 µg/mL). Additionally, the cells treated with leaf extract (20 µg/mL for HeLa and 15 µg/mL for DU145) were kept as positive control and untreated cells were used as controls. The treated and untreated cells after incubation were washed with PBS and fixed with 3.7% paraformaldehyde. Thenafter, DAPI (0.5 µg/mL) was added and incubated for 15 min (dark) followed by washing with PBS. The nuclear morphology of all doses of the treatments and controls were visualized under the fluorescence microscope (EVOS™ M5000, Thermo Fisher Scientific, Carlsbad, CA, USA) with a magnification of 20× equipped with camera. The number of cells expressing apoptotic features was seen as blue fluorescent cells. The experiments were carried in three independent repeats and the results were represented as mean ±SD. Statistical analysis were done using one-way ANOVA followed by Dunnett’s post hoc test with GraphPad Prism software (GraphPad Software, Carlsbad, CA, USA).

#### 3.6.4. In Vitro Wound Healing Scratch Assay

The in vitro wound healing scratch assay is the standard and effective method to investigate and measure cell migration of both cancer cell lines upon treatments [87,88]. This was employed to calculate the rate of migration of the prepared ZnO NPs on HeLa and DU145 cells in comparison with the leaf extract (20 µg/mL for HeLa and 15 µg/mL for DU145) treated cells (positive controls) and negative untreated controls. The measurements were generally taken for 24 h treated cells in an attempt to minimize the contribution of cell proliferation to fill the gap. For the purpose, HeLa and DU145 cells (2 × 10^5^ cells/well) were plated in 6-well plates and incubated in CO_2_ incubator for 24 h to grow in a monolayer and reach 80% confluence. A scratch was made by holding a sterile p10 micropipette tip vertically to scratch a cross in each well of both treatments and controls in both studied cell lines. The suspended floating cells were removed by washing the cells with PBS to ensure that the edges of the scratched surface are clean. Care was taken to ensure the same-dimensional wounds were inflicted in both the cells of treatments and controls to eliminate any potential variation arising from a scratch width difference. After scratching, the cells were reincubated with the doses of ZnO NPs (5 and 15 μg/mL separately) and, the positive and negative control samples contained the respective cells without ZnO NPs and leaf extract. Migration of controls and treated cells were assessed by monolayer gap closure and measured by free ImageJ software (version 1.50i, National Institute of Health, Bethesda, MD, USA). The area of the initial migration of all tested and control cells were measured at 0 h just after the scratch followed by gap area measurements after 24 h of treatments in both cell lines. The scratch closure were observed under fluorescence microscope (EVOS™ M5000, Thermo Fisher Scientific, Carlsbad, CA, USA) with a magnification of 4× and the images were captured in both treatments and controls. The migration percentage was given as the value of gap area to the initial scratch area. Each experiment is individually repeated for three times and the statistical significance was considered at *p* < 0.05.

## 4. Conclusions

*C. verrucosa* is reported to show cytotoxic and anticancer activity and the ZnO NPs synthesized using its leaf extract was found to be more effective than sole leaf extract treatment. Nanosized ZnO of size 16–38 nm was synthesized biologically using *C. verrucosa* leaf extract and was characterized to determine its unique properties that consequently displayed potent antibacterial activity against the studied MDR human pathogenic bacteria. Further, cytotoxicity assay and its visualization results revealed it as a selective substitute for cancer therapy specifically against cervical and prostate cancer with its selective targeting property within the cell. However, there is a need to explain further the exact mechanism of inhibitory action in microbes and the studied cancer cells due to *C. verrucosa* mediated ZnO NPs.

## Figures and Tables

**Table 1:** Antimicrobial activity of green synthesized nanoZnO.

S. No.	Tested Pathogens		Zone of Inhibition (mm) Volume of Nano-ZnO Suspension			Streptomycin
		Negative Control	25 µg/mL	50 µg/mL	100 µg/mL	20 µg/mL
1	*E.coli*	0.0	9 ± 0.2	11 ± 0.0	15 ± 0.9	16 ± 1.7
2	*P. vulgaris*	0.0	11 ± 1.2	14 ± 1.0	17 ± 1.1	18 ± 1.4
3	*K. pneumoniae*	0.0	10 ± 0.4	13 ± 0.5	17 ± 0.5	20 ± 1.2
4	*S. aureus*	0.0	11 ± 0.3	14 ± 0.4	18 ± 0.3	21 ± 0.9
